# Biomarkers of Temporomandibular Disorders: A Narrative Review

**DOI:** 10.7759/cureus.93390

**Published:** 2025-09-28

**Authors:** Maneesha Achuthan PK, Sarika K, Ajith Vallikat Velath, Sapna Varma NK

**Affiliations:** 1 Orthodontics and Dentofacial Orthopedics, Amrita School of Dentistry, Amrita Institute of Medical Sciences, Kochi, IND

**Keywords:** biomarkers, serum biomarkers, temporomandibular disorder, temporomandibular joint management, temporomandibular joint-tmj

## Abstract

Temporomandibular disorder (TMD) refers to musculoskeletal conditions that affect the temporomandibular joint (TMJ) and surrounding tissues, often leading to pain, impaired function, and a diminished quality of life. Despite its clinical relevance, diagnosing and managing TMD remains complex due to its multifactorial etiology. Biomarkers, which are quantifiable indicators of biological processes, have gained attention as valuable tools for enhancing TMD diagnosis, prognosis, and treatment monitoring. Various categories of biomarkers, such as molecular, genetic, sensory, and neuroimaging markers, have been investigated in TMD studies. Biomarker assessment typically employs non-invasive methods, including analysis of saliva, serum, or synovial fluid, and advanced imaging techniques. The incorporation of these biomarkers into clinical practice offers the potential for earlier diagnosis, more accurate prediction of treatment responses, and the development of individualized therapeutic strategies.

## Introduction and background

Temporomandibular disorders (TMDs) comprise a spectrum of conditions affecting the temporomandibular joint and associated structures, presenting with symptoms that range from mild discomfort to chronic pain and significant functional impairment [1]. It affects approximately 30-34% of the global population, with a higher prevalence in women and a peak incidence in early to mid-adulthood [2]. Clinically, patients often present with preauricular pain, joint sounds, restricted or deviated jaw movements, and associated complaints such as headache, otalgia, or cervical discomfort [3]. TMDs are broadly classified into pain-related conditions, such as myalgia, local myalgia, myofascial pain, myofascial pain with referral, arthralgia, and headache attributed to TMD, and non-painful joint disorders, which include disc displacement with or without reduction [4]. The differential diagnosis of TMDs includes dental, otologic, and neurologic disorders, requiring careful history, examination, and selective imaging for accurate distinction [5]. Recently, biomarkers have emerged as promising adjunctive tools to aid diagnosis, phenotyping, and prognosis of TMDs. A biomarker is an objectively measured characteristic that serves as an indicator of normal physiological function, pathogenic processes, or a response to a therapeutic intervention [6].

This review explores studies on biomarkers, such as molecular, genetic, sensory, and neuroimaging markers, and their contribution to advancing personalized healthcare approaches for TMDs.

## Review

Methodology

Search Strategy

A comprehensive literature search was undertaken to identify studies on biomarkers related to temporomandibular disorders. The databases PubMed/MEDLINE, Scopus, and Google Scholar were searched to ensure wide coverage of the available evidence. To improve methodological strength, the search placed emphasis on systematic reviews while also considering meta-analyses, randomized controlled trials, cohort studies, cross-sectional studies, and narrative reviews.

The strategy included combinations such as "Temporomandibular disorders" AND "Biomarker" OR "Molecular biomarker" OR "Diagnostic biomarkers" OR "Predictive biomarkers" OR "Prognostic biomarkers" OR "Safety biomarkers" OR "Genetic biomarkers" OR "Cytokines."

Inclusion Criteria

Articles written in the English language published from 2000 to 2025 were included. The search prioritized studies on molecular, diagnostic, prognostic, predictive, safety, and genetic biomarkers of temporomandibular disorders. Eligible study designs included systematic reviews, meta-analyses, randomized controlled trials, cohort studies, and narrative reviews.

Exclusion Criteria

Articles were excluded if they were not published in English, not peer-reviewed, or published before the year 2000. Additionally, case reports, expert opinions, and editorials that lacked original data were not included. Studies that were unrelated to temporomandibular disorders or lacked a systematic methodology were also excluded.

Biomarkers

A biomarker is an objectively measurable indicator used to reflect biological or pathological conditions and responses to treatment, quantifiable across various biological levels. It offers insights into health status, disease risk, and treatment response through testing body fluids or tissues [6]. Biomarkers have advantages like objective evaluation, reliability, and consistency, making them valuable in studying disease mechanisms and tracking risk [7]. However, they come with challenges, including high costs, timing sensitivity, sample storage issues, laboratory error potential, and ethical considerations.

Types of Biomarkers

The FDA-NIH Biomarker Working Group classifies biomarkers based on their primary clinical use into six categories: diagnostic, prognostic, monitoring, pharmacodynamic/response, predictive, and safety biomarkers. Each type provides specific insights into diseases or treatments, aiding in improved diagnosis, prognosis, and clinical outcomes [8].

Diagnostic Biomarkers

Diagnostic biomarkers are used to identify, detect, or confirm a disease and can also help categorize it or distinguish between different patient variations. However, it's crucial to assess its suitability in research or clinical settings, as it may not always be accurate. Examples of diagnostic biomarkers include interleukin-6, elevated in synovial fluid of internal derangement and degenerative joint disease [9], and C-reactive protein, which shows significantly higher salivary levels in internal derangement [10].

Prognostic Biomarkers

Prognostic biomarkers help to predict the likelihood of disease progression, such as worsening or improvement, in patients. It plays a key role in clinical trials by aiding in the selection of high-risk populations and assessing potential adverse outcomes. These biomarkers guide patient evaluation while avoiding unnecessary procedures or treatments. Examples: matrix metalloproteinase, associated with degenerative joint disease and predicts joint structural deterioration [11].

Monitoring Biomarkers

Monitoring biomarkers is repeatedly measured to track disease progression or response to treatment. It is essential for evaluating pharmacodynamic effects, recognizing early indicators of a therapeutic response, and identifying complications associated with diseases or therapies. Examples include interleukin-8, in synovial fluid levels monitored in disc displacement; higher levels correlate with inferior surgical outcomes [12].

Pharmacodynamic Biomarkers

Pharmacodynamic, or response, biomarkers are used to indicate biological responses following treatment or exposure. These biomarkers play a critical role in clinical practice and early therapeutic research, often serving as endpoints in proof-of-concept studies and aligning with the experimental therapy's mechanism of action. Inflammatory cytokine reduction after treatments (e.g., hyaluronic acid, laser therapy) shows a biological response in chronic TMJ pain [13]. Matrix metalloproteinase levels in saliva change in response to treatment in temporomandibular joint osteoarthritis [14].

Predictive Biomarkers

Predictive biomarkers help to assess a medication’s efficacy or toxicity prediction based on individual biological traits. They are valuable in clinical trials and patient care, aiding in selecting the most suitable treatment for each patient. These biomarkers can also identify risks related to environmental exposures, enabling targeted interventions for at-risk groups. Examples include catechol-O-methyltransferase (COMT) gene variants, which predispose to chronic myofascial pain and TMD and predict poor treatment response [15].

Safety Biomarkers

A safety biomarker is a biomarker that indicates the likelihood of adverse effects due to toxicity in medical products or environmental agents. These biomarkers can detect adverse effects from drug administration or exposure, allowing for dose adjustments or treatment interruption before severe toxicity. Regular monitoring of biomarkers is essential for managing potential toxicity effectively. Examples include liver enzymes, monitored during chronic pharmacologic management of myofascial TMD, particularly with long-term nonsteroidal anti-inflammatory drug (NSAID) use [16].

In TMDs, these biomarkers serve complementary roles: diagnostic biomarkers establish disease presence, prognostic biomarkers predict progression, monitoring biomarkers assess disease activity, pharmacodynamic biomarkers reflect therapeutic response, predictive biomarkers guide treatment selection, and safety biomarkers detect adverse effects, collectively advancing precision and personalized care.

Biomarkers of Temporomandibular Disorders

TMJ biomarkers provide valuable data on the joint's physiological or pathological state, assisting in diagnosis, monitoring, and the formulation of treatment strategies. Table 1 summarizes the types of biomarkers associated with TMDs.

**Table 1:** Classification of Biomarkers in Temporomandibular Disorders

Type of Biomarker	Example
Molecular biomarker	A. Cytokines
	Interleukins (IL)
	Tumor necrosis factor (TNF)
	Matrix metalloproteinases (MMPs)
	B. Neurotransmitters
	Bradykinin
	Prostaglandin (PGE2)
	Glutamate
	Serotonin
	Dopamine
	Substance P (SP)
	Neuropeptides
	Growth factors
	C. MicroRNA
Neuroimaging biomarker	Thalamic Grey Matter Volume (GMV)
Neurochemical biomarker	N-acetyl aspartate (NAA)
	Total creatine (tCr)
	Choline (Cho)
	Myo-inositol (MI)
	Glutamic acid (Glu)
Genetic biomarker	Catechol-O-methyltransferase (COMT)
	Serotonin transporter gene (5-hydroxytryptamine, 5-HT)
	Estrogen Receptor 1 (ESR1)
	Methionine Synthase Reductase gene (MTRR)
	Adrenoceptor Beta 2 gene (ADRB2)
Sensory biomarker	Transient receptor potential vanilloid (TRPV4) Calcitonin gene-related peptide (CGRP)

Molecular Biomarker

Molecular biomarkers are specific molecules, such as cytokines, tumor necrosis factor, and prostaglandins (PGE2), that indicate inflammation and pain in the temporomandibular joint. Blood serum is the primary method for studying molecular biomarkers, but alternatives like saliva and synovial fluid are gaining attention for their non-invasive collection methods and potential specificity to joint health, making synovial fluid analysis valuable for real-time monitoring of joint conditions. Researchers have found molecular biomarkers in chronic TMD conditions [17].

Cytokines

Cytokines, small proteins, are secreted by cells in response to inflammation, primarily by monocytes and macrophages. They modify synovial fluid viscosity, hinder cartilage nourishment, and promote proteoglycan breakdown. Cytokines include interleukins, TNF, and MMPs. Elevated proinflammatory cytokines are linked to symptoms of TMDs, as they break down cartilage and bone in joints. A study by Segami and Miyamaru found a correlation among the pro-inflammatory cytokine concentrations in the synovial fluid of individuals, suggesting the potential for coordinated activation of these cytokines in TMDs [18].

Interleukins

Interleukins, a group of cytokines, play a significant role in the inflammation and pain associated with TMDs.

IL-1: The IL-1 system consists of 21 molecules, forming receptors, co-receptors, ligands, and antagonists. It comprises three legends: IL-1β, IL-1ra, and IL-1α. IL-1β and IL-1α stimulate inflammation, while IL-1RA inhibits it. Interleukin-1β, a proinflammatory cytokine, is linked to inflammatory and degenerative processes in fibroblast-like synoviocytes (FLS), contributing to rheumatoid arthritis. Increased IL-1β levels in synovial fluids of individuals having bone arthritis and joint conditions affect the TMJ, while IL-1ra levels decrease in inflammatory arthritis [19]. Ogura et al.’s study, using an oligonucleotide microarray approach, found that synovial fibroblasts, triggered by IL-1β, release chemokines, indicating their role in initiating and developing TMJ inflammation [20].

IL-6: It contributes to the progression of temporomandibular joint disorders and is an important pro-inflammatory cytokine. It has a vital role in the growth and maturation of immune cells, for example, neutrophils, macrophages, cytotoxic T-lymphocytes, and natural killer cells. Interleukin-6 level was greater in patients with degenerative joint disease with disc displacement. The plasma levels of IL-6 can be detected within an hour of tissue injury [21,22].

IL-8: It, mainly produced by macrophages, is crucial for inflammatory reactions and is involved in diseases like rheumatoid arthritis, contributing to neutrophil infiltration and joint inflammation. Hypoxia can enhance IL-8 production. Higher levels of IL-8 are found in the synovial fluid of patients who have TMJ internal derangement (ID), particularly at the posterior disk attachment [21,23].

TNF

TNF, a cytokine, regulates immune responses and inflammation. It is produced by various cells and promotes inflammatory mediators. TNF is associated with TMDs, pain perception, tissue damage, and inflammation within the TMJ complex. Elevated TNF levels in TMDs suggest its involvement in the pathophysiology. Elevated TNF levels in peripheral afferent fibers and dorsal root ganglion neurons are linked to peripheral pain mechanisms. Patients with mandibular movement-evoked joint pain have higher TNF levels in synovial fluid. Glucocorticoid injections reduce TNF levels and provide pain relief. TNF's sensitivity in pain inflammatory diseases can help evaluate pain mechanisms and track its involvement in painful TMJ disorders [24,25].

Matrix Metalloproteinases

MMPs take part in tissue degradation along with inflammation, playing a role in temporomandibular joint inflammation and pain modulation. They are triggered by normal and abnormal physiological functions. Joint inflammation increases the production of genes like MMP-13, MMP-1, and MMP-2 that are involved in matrix breakdown. Huang X and colleagues found that MMP-3 expression is linked to TMJ lesions in rats. Nascimento et al. found changes in MMP-2 and MMP-9 expression during joint inflammation. MMPs are being explored for pain management [26,27].

Neurotransmitters

Neurotransmitters are crucial for sensitization and central processing. They significantly impact mood, behavior, and pain transmission. Major neurotransmitters include bradykinin, PGE2, glutamate, NGF, substance P (SP), serotonin, histamine, leukotriene B4 (LTB4), norepinephrine, inflammatory mediators, and calcitonin gene-related peptide (CGRP). These substances are linked to both inhibitory and facilitatory pain processes.

Bradykinin: An inflammatory mediator, it performs a key role in sensitization and nociception. It acts as a bronchoconstrictor and a vasodilator, and it increases vascular permeability, as well as contributing to pain transmission. A recent study investigating bradykinin release in TMJ internal derangement and degenerative joint disease (DJD) found a direct relationship between higher bradykinin levels and the severity of the inflammatory response [28].

Prostaglandin: PGE2, produced by the prostaglandin cyclooxygenase (COX) enzyme, is a significant pain biomarker in chronic inflammatory joint disease. Its levels in synovial fluid correlate with mandibular movement-induced TMJ pain. Basi et al. found that pain mediators, except F2-isoprostane, significantly correlate with muscle, synovial fluid, and plasma, along with painful TMD [29,30].

Glutamate: The most prevalent excitatory neurotransmitter in afferent sensory neurons is glutamate. The central nervous system receives sensory signals from it. Normal sensory neurons have glutamate receptors, for example, glutamate-N-methyl-D-aspartate (NMDA), along with alpha-amino-3-hydroxy-5-methyl-4-isoxazolepropionic acid receptor (AMPA). TMD patients experience peripheral and central sensitization due to elevated glutamate levels in joints, which are associated with inflammatory diseases like rheumatoid arthritis. Increased glutamate levels in the temporomandibular joint's synovial fluid during TMD add to chronic pain and increased sensitivity linked to TMD. The NMDA receptor pathway is particularly active in TMD, resulting in more severe and persistent pain sensations. Glutamate injection into the TMJ causes pain within seconds, partially regulated by peripheral NMDA receptors [31].

Serotonin: 5-HT, which is also referred to as serotonin, controls physiological nervous system functions. Serotonin is found in central nervous system (CNS) serotonergic neurons, affecting pain modulation. Serotonin as a biomarker in hyperalgesic patients. A study found higher serotonin levels in TMJ synovial fluid, linked to TMJ pain from mandible functional movements. In the Fredriksson et al. study on 5-HT's role in TMJ pain reduction, 5-HT regulates the efficacy of intra-articular glucocorticoids in inflammatory joint disease. Local and systemic 5-HT levels affect pain relief in chronic arthritis or degenerative joint diseases [32].

Dopamine: TMD pain is influenced by dopamine, a neurotransmitter involved in pain sensation, movement control, information processing, and reward processing. Dawson's study contradicted the hypothesis of a difference in 5-HT plasma levels between patients with myofascial TMD and healthy subjects, confirming an elevation in plasma dopamine levels [28].

Neuropeptides: These are produced by neurons and are involved in the communication and release of neurotransmitters like acetylcholine and norepinephrine. Some neuropeptides, like SP along with CGRP, have a role in neurogenic inflammation and enhance certain inflammatory and nociceptive processes, with their release varying depending on their location. Sato et al. found a direct relationship between CGRP levels and pain in patients with painful TMD. Although SP levels were increased in synovial specimens, no correlation was found with CGRP levels, which were suggested to be more reliable in indicating joint discomfort than SP [33].

Growth factors: NGF is a key modulator of chronic pain, activating signal transduction in peripheral sensory neurons during injury or temporomandibular joint inflammation. This triggers pathways like PI3K-AKT, ERK-phosphorylation, and escalation of protein kinases, amplifying inflammatory pain. A study found elevated serum vascular endothelial growth factor (VEGF) in disc disorders linked to tissue degradation, and other molecules linked to TMJ disorders include insulin-like growth factor binding protein (IGFBP), brain-derived neurotrophic factor (BDNF), fibroblast growth factor (FGF), and transforming growth factor (TGF-alpha) [34].

*MicroRNA: *These small non-coding inhibitory ribonucleic acid (RNAs), approximately 19-24 nucleotides long, regulate pain processing across various models and clinical pain conditions. They attach to target mRNAs' 3′ or 5′ UTRs. Xu et al. found miRNA221-3p levels lower in synovial fibroblasts in degenerative diseases, repressing the Ets-1 transcription factor, linked to MMPs, responsible for joint cartilage tissue deterioration and reconstruction. The role of miRNA 140-5p in TMJ DJD is examined by Li et al., who propose that it may preserve the homeostasis of mandibular condylar cartilage and act as a prognostic indicator for TMJ degenerative alterations. Zhang and colleagues suggested that the degeneration of the cartilage matrix and the emergence of degenerative alterations in the TMJ may be attributed to the functional involvement of miR-101a-3p and miR21-5p [35,36].

Neuroimaging Markers

As TMD progresses, neurons in central pain processing areas and tissue-dedicated nociceptors become more active, potentially serving as biomarkers. Neuroimaging is used to investigate these biomarkers. Moayendi et al. found a direct relationship between GMV in the thalamus and pain duration, suggesting nerve microstructure changes could serve as biomarkers for understanding TMD-related pain mechanisms [37]. A study by Younger et al. on 35 females with myofascial TMD found a notable rise in grey matter volume in the right anterior insula and reduced GMV in the posterior cingulate cortex [38]. According to Zhang's research, female patients with TMJ synovitis pain had lower Rh factor in blood (RHO) values in their right anterior insula and less connection between their anterior insula and median cingulate cortex, which were connected with the degree and severity of their pain [39].

Neurochemical Biomarker

Neurochemicals are related to neuron health, metabolic processes, and osmotic balance. Magnetic resonance spectroscopy (MRS) imaging was used to determine neurochemicals like NAA, tCr, Cho, MI, and Glu. Research suggests that certain neurochemicals can be used to understand TMD pain. Harfeldt et al. discovered increased tCr concentrations in TMD patients' posterior insula, higher Cho values associated with restricted mouth opening capacity and pain pressure threshold, and higher Glu values with augmented temporal summation of non-weight-bearing mechanical stimulus, offering insights into neurochemicals as biomarkers for TMD pain mechanisms [40].

Genetic Biomarkers

TMD's complex nature suggests multiple genetic loci interact with environmental factors, influencing its development and progression. Allelic association methods are most effective for studying frequently occurring variants.

COMT: An enzyme named COMT is responsible for breaking down and retaking catecholamines, for example, norepinephrine, epinephrine, and dopamine. Genetic variations in the COMT gene can reduce enzyme activity, leading to higher levels of catecholamines and lower pain tolerance. Genetic variations in COMT linked to TMD are found in both coding and non-coding regions, including rs4680 and rs4818. Brancher et al. discovered that 4 COMT SNPs (rs4633, rs6269, rs4680, and rs4818) create three primary haplotypes linked to pain, sensitivity, and increased TMD risk [15].

5-HT: Serotonin, a neurotransmitter, is crucial for pain relief. The human serotonin transporter gene (5-HTT), which is found on chromosome 17q11.1-q12, is considered a potential contributor to the development of painful conditions like TMD. In comparison, individuals having the common genotype, those with TMD myalgia who carry the homozygous uncommon or heterozygous genotypes of the HTR2A (rs9316233) and HTR3A (rs1062613) SNPs reported higher pain intensity (CPI) and jaw functional limitation (HTR3A) [32].

ESR1: Painful TMDs in women are largely due to female reproductive hormones, particularly estrogen, affecting inflammation and central pain pathways. The TMJ's synovitis and bone and cartilage deterioration may also be related to the ESR1 gene, which codes for estrogen receptor alpha. Quinelato's study found that the ESR1 and ESRRB genes differed significantly from a control group. Temporomandibular arthralgia and muscular TMDs were found to be much more likely to occur in people with the TT genotype of the ESR1 (rs2273206) gene. For the ESRRB (rs1676303) gene, the CC genotype was associated with the presence of articular TMDs and other chronic arthralgia. Polymorphisms in the ER-α receptor gene, specifically Pvu II (rs2234693) along with Xba I (rs9340799), appear to be connected to osteoarthritis’s various forms, like TMD [41].

MTRR: The MTRR gene, involved in methionine synthase, is linked to TMD pain. A mutation in the gene, rs1801394, alters the Ile22Met, causing a reduced substrate affinity. This variant is particularly prevalent in individuals with polycystic ovary syndrome (PCOS), indicating a potential link to TMD pain [42].

ADRB2: The ADRB2 gene is responsible for coding a protein. Variations in the ADRB2 gene can affect an individual's pain sensitivity. The ADRb2 gene, located on chromosome 5q31-32, is a key gene in managing chronic pain and influencing resting arterial blood pressure. Variations in the rs1042713 region, specifically at codon 16, have been linked to a tenfold increase in the likelihood of developing TMD. The gene contains eight single-nucleotide polymorphisms (SNPs) and three main haplotypes: H1, H2, and H3. H2/H2 homozygotes have the highest incidence of myogenous TMD, suggesting that having a minimum of 1 copy of the H1 haplotype may protect against it [43].

Sensory Markers

TMD pain is significantly influenced by sensory neurons within the trigeminal ganglion, which transmit nociceptive signals from the peripheral nervous system to the central nervous system.

Suttle et al. found that the trigeminal ganglion neurons, which innervate the TMJ, express TRPV4, which increases after joint inflammation. Inhibiting TRPV4 significantly reduced masticatory pain induced by TMJ inflammation. CGRP is a significant pain mediator in joint arthritis and fibromyalgia, with increased levels in synovial fluid linked to pain in arthritic temporomandibular joints. CGRP-receptor antagonism has been reported to reduce TMD-related pain in experimental and clinical contexts. CGRP in peri-TMJ tissues triggers proinflammatory mediators, sensitizing or activating trigeminal nociceptive neurons [44].

Techniques of biomarker analysis

Biofluids

Biofluids are biological fluids used for clinical, laboratory, and prognostic diagnosis and monitoring of patients' health. They contain valuable information for informed diagnosis and treatment, such as detecting biomarkers for temporomandibular disorders. Table 2 lists biofluids and their associated biomarkers identified in TMDs.

**Table 2:** Types of biofluids and their associated biomarkers in temporomandibular disorders

Type of Biofluid	Biomarker
Blood serum	Interleukins: IL-6, IL-1β, IL-8, IL7, IL-13
	Monocyte chemotactic protein 1(MCP-1)
	Prostaglandins (PGE2)
	F-2-Isoprostane
Blood plasma	Growth factors: Nerve growth factor (NGF)
	Vascular Endothelial Growth Factor (VEGF)
	Serotonin
Synovial fluid	Proteinases: Matrix metalloproteinases- MMP-1, MMP-3, MMP-2, MMP-8, MMP-7, MMP-13MMP-9,
	Interleukins: IL-6, IL-1β, IL-8, IL7, IL-13
	Monocyte chemotactic protein 1 (MCP-1)
	Bradykinin
	Glutamate
	Serotonin
	Dopamine
	Neuropeptides: Substance P (SP), Calcitonin gene-related peptide (CGRP)
Saliva	Serotonin
	Glutamate
	MMP-3
	Tumor necrosis factor (TNF)
	Neuropeptides: SP, CGRP
Urine	Pyridinoline (PYD)
	Deoxypyridinoline (DPD)
	C – terminal telopeptide of Type I (CTX-I)
	C – terminal telopeptide of Type II (CTX-II)

Neuroimaging Methods

Neuroimaging methods help identify biomarkers by visualizing brain structure, activity, and metabolism associated with pathological conditions [45]. Table 3 presents various neuroimaging methods used for biomarker identification.

**Table 3:** Neuroimaging methods

Neuroimaging methods	Description
Blood Oxygen Level Dependent Functional Magnetic Resonance Imaging (BOLD fMRI)	Identify brain activity through the levels of oxyhaemoglobin and deoxyhaemoglobin.
Functional Magnetic Resonance Imaging - Arterial Spin Labeling (fMRI-ASL)	The method utilizes water from artery as a natural tracer to assess blood flow in cerebral part of brain.
Magnetic Resonance Imaging -Diffusion Tensor Imaging (MRI-DTI)	Quantifies the water diffusion within the brain. & MRI-Structural - Structural data defines the white and gray matter distribution within voxel-based parameters.
Proton Magnetic Resonance Spectroscopy (H-MRS)	Quantifies compounds bonded with protons, signifying metabolic activity

Quantitative Sensory Testing (QST) in TMD Pain

QST is a psychophysical method used to evaluate the somatosensory system. A study found that QST can identify unique pain patterns in specific TMD cases, potentially aiding in the diagnosis of painful TMD. Research indicates that individuals with higher pain sensitivity are more likely to develop TMD, with somatosensory abnormalities in TMD patients compared to control groups [46].

Future perspectives

Machine learning and artificial intelligence are expected to significantly analyze datasets, identify novel biomarkers, and guide clinical decision-making in TMD pathophysiology, disease onset prediction, and treatment strategies. Another rapidly evolving area of exploration is digital biomarkers. A digital biomarker refers to a quantifiable, objective measure of physiological or behavioral data that is collected through digital devices such as smartphones, wearable sensors, or other digital tools. These biomarkers can be used to monitor, diagnose, and predict health conditions in real time, offering a non-invasive and continuous way to gather health-related data [47,48].

## Conclusions

The study of biomarkers in TMDs holds significant promise for both advancing scientific understanding and improving clinical practices. By pinpointing specific biomarkers related to TMD mechanisms, clinicians can enhance diagnostic accuracy, refine prognosis, and personalize treatment. Furthermore, the identification of consistent biomarkers could lead to the creation of targeted therapies, ultimately improving patient outcomes and satisfaction. However, additional research is essential to verify the effectiveness and consistency of these biomarkers across various TMD types and diverse patient groups. A universally accepted "gold standard" biomarker for TMD is yet to be identified.
